# Report on Use of a Methodology for Commissioning and Quality Assurance of a VMAT System

**DOI:** 10.1371/journal.pone.0058877

**Published:** 2013-03-15

**Authors:** Charles Mayo, Luis Fong de los Santos, Jon Kruse, Charles R. Blackwell, Luke B. McLemore, Deanna Pafundi, Joshua Stoker, Michael Herman

**Affiliations:** Department of Radiation Oncology, Mayo Clinic, Rochester, Minnesota, United States of America; The University of Chicago, United States of America

## Abstract

**Introduction:**

Results of use of methodology for VMAT commissioning and quality assurance, utilizing both control point tests and dosimetric measurements are presented.

**Methods and Materials:**

A generalizable, phantom measurement approach is used to characterize the accuracy of the measurement system. Correction for angular response of the measurement system and inclusion of couch structures are used to characterize the full range gantry angles desirable for clinical plans. A dose based daily QA measurement approach is defined.

**Results:**

Agreement in the static vs. VMAT picket fence control point test was better than 0.5 mm. Control point tests varying gantry rotation speed, leaf speed and dose rate, demonstrated agreement with predicted values better than 1%. Angular dependence of the MatriXX array, varied over a range of 0.94–1.06, with respect to the calibration condition. Phantom measurements demonstrated central axis dose accuracy for un-modulated four field box plans was ≥2.5% vs. 1% with and without angular correction respectively with better results for VMAT (0.4%) vs. IMRT (1.6%) plans. Daily QA results demonstrated average agreement all three chambers within 0.4% over 9 month period with no false positives at a 3% threshold.

**Discussion:**

The methodology described is simple in design and characterizes both the inherit limitations of the measurement system as well at the dose based measurements that may be directly related to patient plan QA.

## Introduction

Designing a comprehensive approach to commissioning and quality assurance (QA) for a volumetric modulated arc therapy (VMAT) system requires consideration of tests addressing delivery of the beam from several perspectives [1]–[5]. Control points for a VMAT beam manage modulation of the position and speed of the multileaf collimator (MLC) leaves, gantry angle, rotation speed, and dose rate as a function of the fraction of total number of monitor units delivered. Control point tests, such as the picket fence, and tests introduced by Ling et al. [1] or tests later expanded upon by Van Esch et al. are a key component of commissioning [2]. In general results from these cannot be connected in a predictive way to dose delivered to a patient, and thus additional measurements become necessary. Commissioning measurements reported by Task Group 119 made initial verification by phantom studies of standardized treatments that can be planned, prepared and delivered with sufficient accuracy [6]. The authors of Task Group 119 used simple and reliable phantom setups to measure dose distributions designed for phantom targets and for patient dose distributions projected onto the target.

One of the tasks of commissioning is designing and carrying out a set of tests that can serve as a baseline for an ongoing QA program. The report of Task Group 142 defines scheduled MLC performance tests for intensity-modulated radiation therapy (IMRT) [7].While TG142 did not explicitly address VMAT, it is reasonable to anticipate the need for a supplemental set of tests targeted at VMAT [3].

In setting up a VMAT Commissioning and QA program, additional details require attention. Limitations of the specific measurement devices used and influence of the treatment couch on the measurements must be addressed [8]–[12] It is clinically desirable to be able to treat patients with both full and partial arcs (spanning a small asymmetric angular range) using the full range of gantry angles. This includes those gantry angles that result in beams intersecting the couch; therefore influence of the treatment couch on dose, along with limitations of the specific devices used for measurements need to be addressed.

The measurement methodology used for commissioning should align with that used for routine patient QA measurements. Design of standardized control point tests facilitated their use in multiple institutions and benchmarking of results, similarly it is also desirable to design phantom dose measurements that can be used as a benchmarking standard as well. Additionally, routine QA tests, with corresponding tolerance and action levels, should be designed for simple and reliable use. This report introduces an approach used within our facility to address these issues.

## Materials and Methods

Commissioning was performed on a Varian Linac 21EX with 120-leaf Millennium MLC equipped with an image-guided radiation therapy (IGRT) couch. Control point tests, developed by Ling et al., were carried out [1]. Standard Digital Imaging and Communications in Medicine (DICOM) radiation therapy plan (RT) files, used to control the accelerator for the tests, were downloaded from the MyVarian website. Measurements were carried out using portal dosimetry. The first test compares a static-gantry picket fence pattern with a 5 mm slit, to one delivered with gantry rotations from 179° to 187°. The second test uses seven dose-rate and gantry speed combinations (105, 209, 314, 419, 524 and 589 MU/min at 4.8°/sec, 600 MU/min at 4.4°/sec within 2-cm wide bands of a 15 cm × 20 cm field. The bands are designed to deliver the same relative fluence pattern at the center of the band as an open field at the corresponding position. The third test uses four leaf speeds (1.67, 2.5, 0.83 and 0.42 cm/sec) within 3 cm wide bands of a 12 cm ×20 cm field. Parameters for constancy tests 2 and 3 differed from the ones described by Ling et al. in order to allow delivery of the test within the operating parameters of a clinical system. These tests are repeated on an annual basis as part of the routine QA program.

In addition, phantom dose measurements were also designed to carry out end-to-end testing, i.e. from simulation to treatment using the same data transfer and treatment mechanisms used for patients. The device used for phantom measurements was scanned in the clinical CT scanner. Treatment plans were designed using a commercial treatment planning system (Eclipse, v 8.9) and transferred to the radiation oncology information system (ROIS) (MOSAIQ, v 1.6) following the routine patient treatment process. Plans were transferred from the ROIS to treatment unit, where the phantom was then treated and dose measurements acquired. In this way all components of the treatment workflow were checked.

Several objectives were used to define standardized target volume to contour and optimization parameters. The target volume should facilitate benchmarking against delivery approaches, demand sufficient optimization, provide for simple and reliable setups, and be accessible to the community at large. Phantom targets that simulate patient target volumes are often used, but they generally do not facilitate benchmarking the accuracy of the measurement system against simple, fixed, open beams. While a spherical volume results in an IMRT pattern with the leaves primarily at the edge of the target, a cubic volume with optimization constraints, forcing a high degree of conformality and uniformity, is satisfactory. The resulting dose distribution is similar whether treated with simple four field box (FFB), five-field IMRT or two VMAT beams approach. The resulting dose gradient is low, both in-plane and out-of-plane, making the test relatively insensitive to alignment. Additionally, a cubic target can be easily constructed in most modern commercial treatment planning systems and is easily scaled to examine target volume effects.

**Figure 1:**
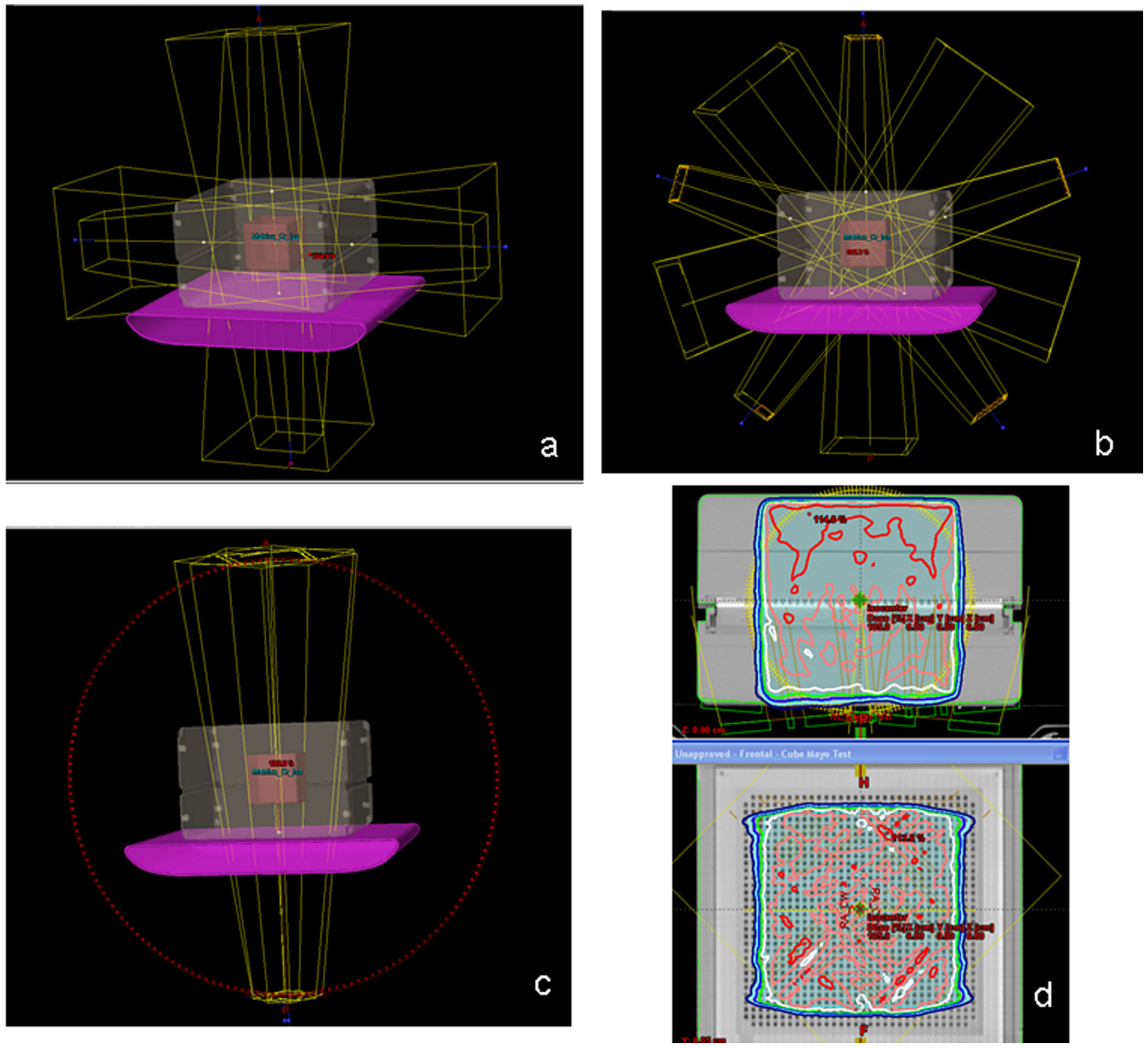
Phantom measurement geometry utilized a cubic target (10 or 20 cm on a side) treated with a) un-modulated four field box, b) five field IMRT and c) two arc VMAT plans, including the couch as a structure. Transverse and coronal views of the dose given to the 20 cm target, as calculated by the treatment planning system, are illustrated in d.

We elected to routinely carry out patient-specific QA measurements at the corresponding treatment delivery gantry angles in both the coronal and sagittal planes. This makes the treatment couch an intrinsic part of the measurement system, (figure 1) The standard Varian IGRT couch supporting structure was used in the treatment plan with the CT numbers for couch surface and couch interior of −550 HU and −950 HU, correspondingly. Measurements carried out for the FFB provide a benchmark of the intrinsic accuracy possible for the measurement system without intensity modulation. Cubic targets with 20 and 10 cm on a side were used.

Typically, measurement systems show some degree of angular dependence, although the magnitude may vary significantly between systems and incident angle. This sensitivity should be characterized, and, if possible, corrected for in the measurements of the dose distribution. This limits uncertainty in ability to generalize commissioning results to an arbitrary weighting of patient beam angles. With the cubic phantom and target volume, the beams of the FFB treatment represent common clinical extremes on the angular dependence. Measurements were carried out to demonstrate agreement with predicted doses for the individual beams as well as for cumulative dose.

In addition to the phantom study, calculated and planned dose distributions were compared for five simple and nineteen complex plans in both the sagittal and coronal planes for a total of 48 measurements. Partial arcs were used for one of the simple plans and ten of the complex plans. Calculations of gamma were carried out for the criteria 3%/3 mm versus 4%/4 mm using a threshold of 10% maximum dose. Results were compared, with and without angular correction.

### Device Specific Angular Correction

The dose (D) at point (x, y) in the phantom, for plan (p) is calculated with the treatment planning system as the sum over the angles of incidence. This is proportional to the product of the response (R) per monitor unit (MU) of the detector at the point in the phantom, the delivered MU, and a correction factor (Φ) that compensates for the relative angular dependence of the R at each point in the detector. A calibration factor (C) scales the product to dose for a defined calibration setup
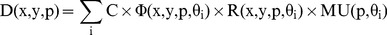
(1)


The angular correction factor, Φ, may depend on plan parameters such as field size. Since Φ cannot be known a priori, it must be measured relative to a calibration condition. If a calibration condition corresponding to an extreme in Φ is selected, e.g. irradiation at a single angle where Φ is at its maximum value, an over-emphasis would occur in subsequent patient measurement errors at angles where Φ is at its minimum. To avoid this issue, we selected a calibration condition similar to patient plan measurements. To calibrate, the phantom is irradiated with a simple 10×10 arc field (179.5° to 180.5°), with a specified number of monitor units MU_cal_, delivering a dose, Dose_cal_, to the isocenter.
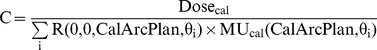
(2)


Since the basis of per patient QA measurements is demonstration of agreement between calculated and measured dose distributions, when a new version of planning system software is installed, it is necessary to confirm that the calibration factor is not affected. Incremental improvements in the software, such as accounting for the treatment couch or heterogeneities, can affect the calculated dose used in determination of the calibration factor, C. In the specified geometry, calculated in Eclipse v8.9 Dose_cal_ = 189.7 cGy for MU_cal_ = 300 MU.

A series of measurements were carried out to characterize Φ, defined as the ratio of measured and predicted doses for a set of simple square field plans irradiating the detector plane at angles θ_i_.
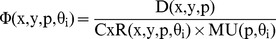
(3)


The device used in these measurements, MatriXX (IBA Dosimetry, Bartlett, TN), is a 24.4. × 24.4 cm^2^ array of 1,020 vented cylindrical ion chambers. The chambers measure 4.5 mm in diameter by 5 mm tall and are spaced every 0.762 cm over the area of the array. The rows and columns of chambers are symmetrically offset from the center by 0.381 cm, so that no chamber is located at the center of the array. The MatriXX array was used within the MultiCube phantom (IBA Dosimetry), which is a modular block of PlasticWater (CIRS, Norfolk, VA). The block consists of several layers that can be arranged to place the MatriXX array at various depths within the phantom. We used a single configuration, with a total thickness of 21 cm for the phantom, and the MatriXX array in the center of the block. The MultiCube phantom was scanned twice with our GE LiteSpeed® CT scanner – once with the phantom placed flat on the table so that the MatriXX array was in a coronal plane, and again with the phantom tipped on its side so that the array occupied a sagittal plane.

Each CT scan was transferred to our treatment planning system (Eclipse v. 8.9, Varian Medical Systems, Palo Alto, CA). A large number of treatment plans were calculated with the MatriXX array in the coronal plane, centered at the LINAC isocenter. All plans consisted of a single open field, 27×27 cm^2^, with 200 MU per field. This field size illuminates the entire detector array without irradiating sensitive electronics mounted nearby in the MatriXX device. The couch supporting structure was included during the treatment planning process. Each plan irradiated the array from a different gantry angle starting at zero, in five degree increments, all the way around the device. Additional plans were added at gantry angles of 2°, 92°, 178°, 182°, 272°, and 358° for a total of 80 calculated dose planes. These dose planes were exported from Eclipse in DICOM and stored for comparison with measured planes.

The MultiCube phantom, with the MatriXX array, was then placed on the treatment unit couch and aligned to isocenter. Each of the eighty planned fields was delivered to the MatriXX at their respective planned gantry angles. An inclinometer connected to the MatriXX enabled recording the gantry angle with the measurement. Measurements for all fields were recorded in ‘Movie Mode,’ in which ion chamber signals are periodically read and stored as ‘snap’ images. At the end of a measurement the readouts are stopped manually and the snap images summed into a total dose plane. These measurements were performed with a 1-one second snap image readout period, so each field measurement, at each gantry angle, was made up of approximately fifty snap images.

Each of the acquired dose planes were then compared to the calculated planes using IBA software (OmniPro-I’mRT v 1.7 b). A measured plane was registered to its corresponding calculated dose plane, and the IBA software calculated a ratio, Φ(x, y, p, θ_i_) between the two dose planes. The dose ratio was then exported to a text file on a grid that matched the dimensions and spatial resolution of the measured plane. The dose ratio tables for all eighty fields were then concatenated into a single angular correction file. Angular correction for the sagittal plane was offset by 90° from the coronal plane. 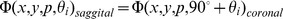
(4)


### Routine QA Test

**Figure 2:**
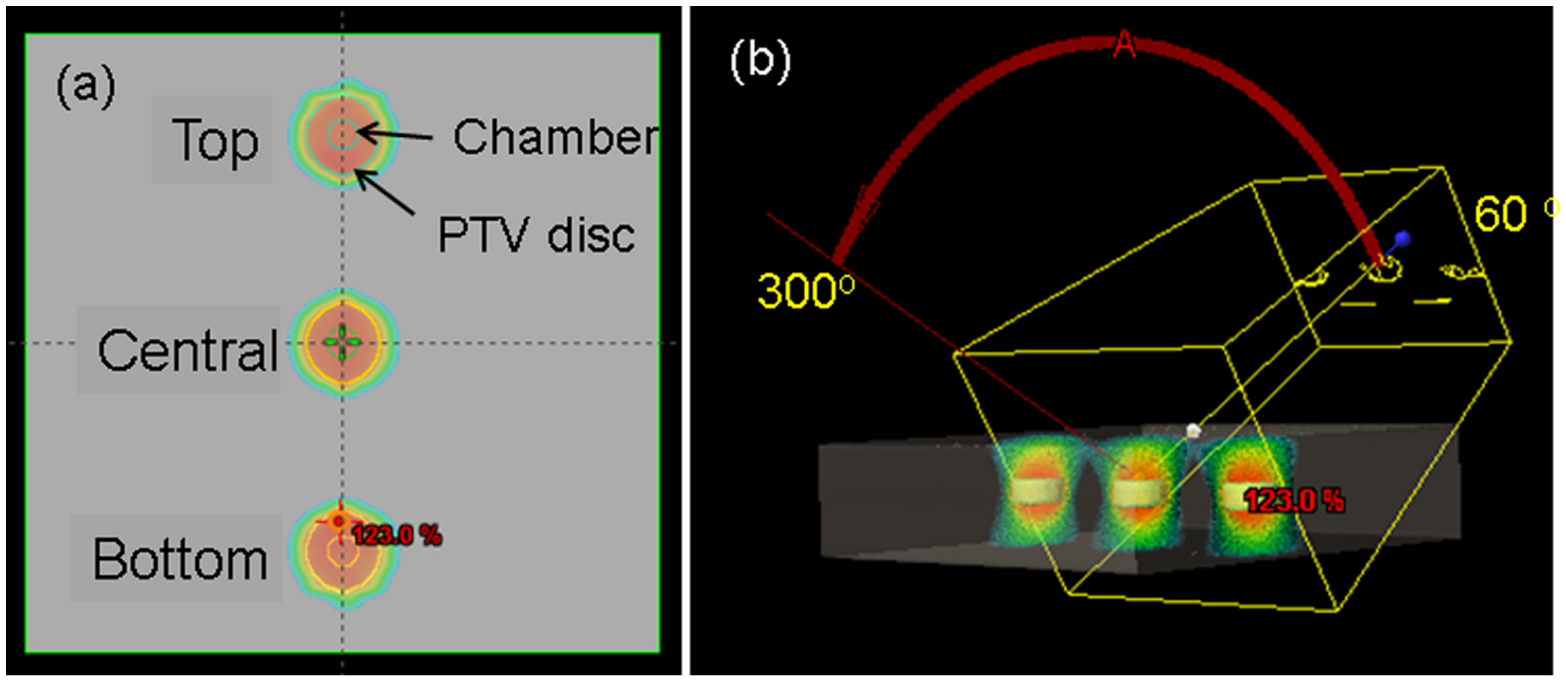
Model of a daily QA device on the treatment planning system. The three chambers were contoured with a PTV expansion of 1 cm on the circumference and 0.5 cm ant-to-post. The VMAT plan delivers a homogeneous dose distribution (200 cGy) at each chamber using a 6X beam moving from 60° to 300°.

In our practice, the TRACKER (model 90100) is used for the daily QA of output, symmetry, and flatness. The detector contains a centrally located ion chamber with four orthogonally placed ion chambers within a 10 cm radius from the central chamber (active cross-sectional area: 0.93 cm^2^). The device was modeled in the treatment planning system with a PTV ellipsoid having a 1 cm margin sup-to-inf and left-to-right, and 0.5 cm ant-to-post from each of the three chamber volumes along the Y axis. This assured minimal sensitivity to setup uncertainties of the device. PTV. The measuring system (figure 2) was set up at a SSD of 100 cm with 3 cm solid water build up. A single 6X, arc spanning 120° (from 60° to 300°) was planned to deliver a homogeneous dose distribution of 200 cGy to the each chamber with 266 MU (figure 2b). This configuration was selected to provide modulation of the three VMAT components: the MLC speed, the dose-rate and the gantry speed during the delivery of the field. The central axis chamber is irradiated using modulation of the 0.5 cm leaves and the outer chambers using a combination of 0.5 cm and 1 cm leaves.

## Results

**Figure 3:**
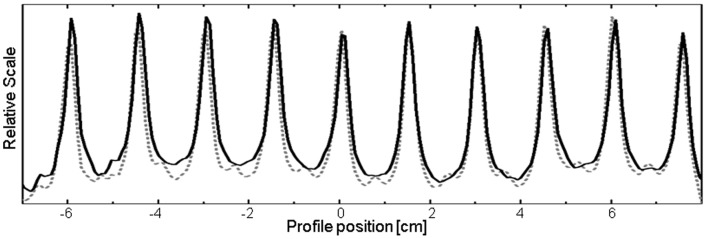
Comparison of DMLC accuracy positioning between fixed-gantry (dashed) and VMAT(solid) picket fence field delivery. Difference in location was ≤0.5 mm for each of the 10 pickets.

**Figure 4:**
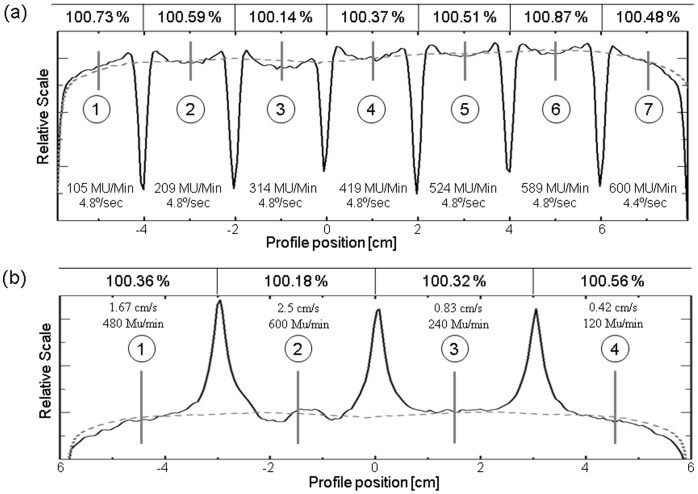
Summary of control point tests on a) dose rate-gantry speed and b) leaf speed-dose rate plotting VMAT (solid) versus open field (dashed). Fluence agreement was within 0.87% and 0.56% for dose rate – gantry and leaf speed-dose rate, respectively.

Control point testing results revealed overall acceptable VMAT system performance. The static versus VMAT pickets (figure 3) demonstrated ≤0.5 mm agreement in peak location. Test results for gantry and dose rate variation (figure 4a) were within 0.87% of the intended values, and results for leaf speed variation (figure 4b) were within 0.56%.

**Figure 5:**
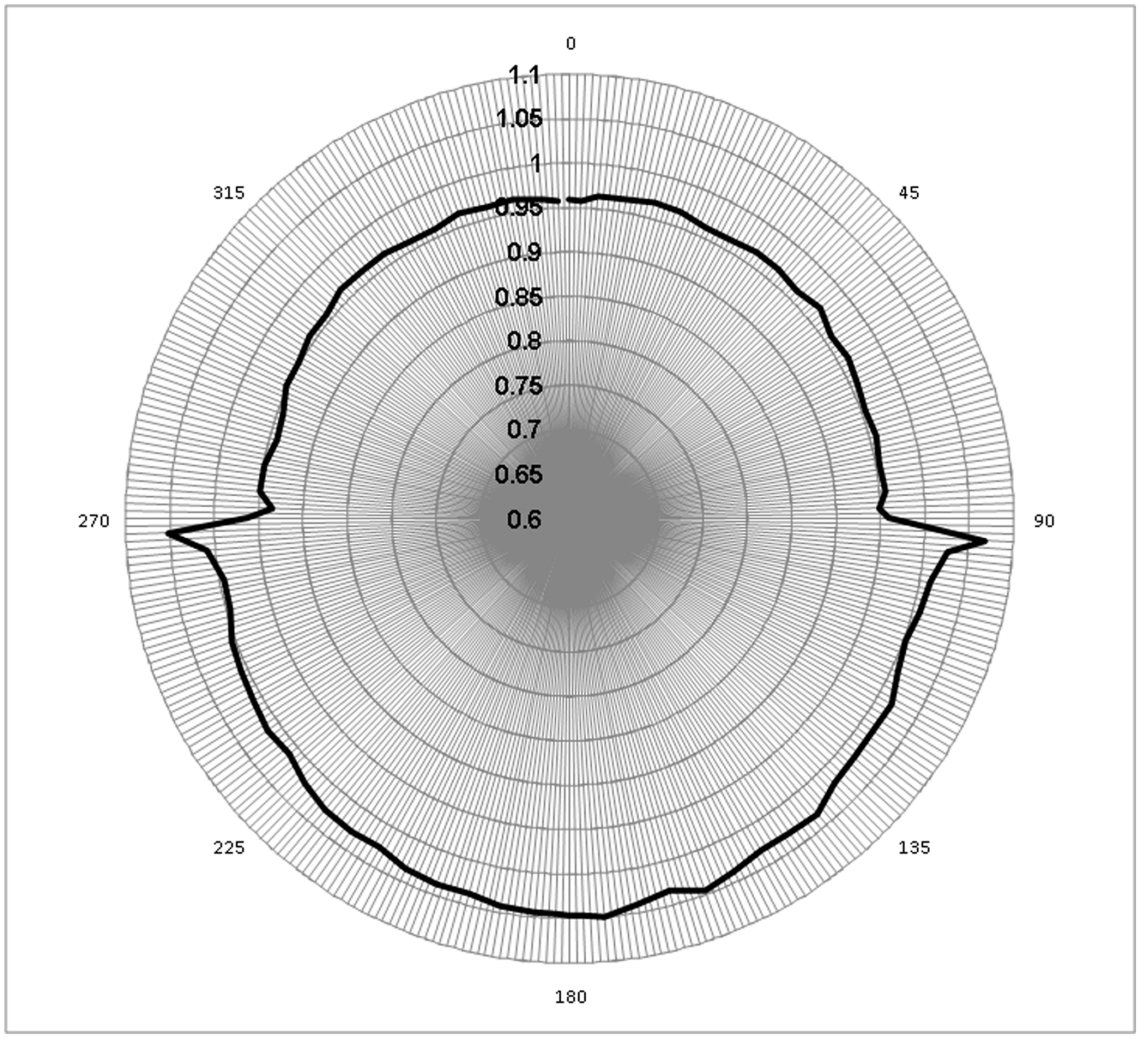
Mean angular correction value for all chambers in the detector array vs. gantry angle.

Angular dependence of the MatriXX response was significant (figure 5). Values for Φ, the angular correction factor, ranged from 0.945 to 1.06 with large variation (∼0.03/degree) at 90° and 270°. Since couch attenuation was included in the plan, the difference between anterior and posterior angles was dominated inherent support structure of the MatriXX. Use of Φ generally improved agreement with calculated doses by 1.7%, 1.25%, and 0.55% for total FFB, IMRT and VMAT dose, respectively. VMAT fields had better central axis dose agreement with the predicted calculation than IMRT or FFB.

The dose at the center of the array was calculated using an average over the four central chambers of the MatriXX for each of the plans, with and without application of the angular correction Φ (table 1). Agreement over the target area was checked by measuring γ(dose deviation/distance to agreement, signal threshold), with analysis parameters of γ (2%/2 mm, 80%) and γ(3%/3 mm, 80%) (table 2). Use of an 80% threshold of maximum dose focuses attention on the central, high dose regions where γ is dominated by dose differences. Also increasing threshold dose values has been shown to produce lower passing rates, giving rise to stricter tests^(11)^. Since the average angular response of the chambers is near unity, the difference in response over the detector matrix is small for plans with beams symmetrically spaced around the target. However, patient plans often use non-uniform arc spacing of beams to spare normal tissues. This can result in substantial differences for plans heavily weighted from posterior or lateral angles. The un-modulated FFB plans demonstrate intrinsic accuracy for measuring central axis dose on the order of 1% and 2.5%, with and without Φ, respectively. FFB measurements for individual angles suggest the potential limitation in measurement accuracy for plans heavily weighted from lateral beam angles if Φ is not used.

**Table 1:** Central Axis dose agreement between measurement and calculation.

Plan	Target	Dose Summation	No Φ	With Φ
FFB	10×10	Total	2.3%	0.5%
		Gantry 0°	3.3%	0.0%
		Gantry 90°	3.4%	1.0%
		Gantry 180°	−5.2%	−0.1%
		Gantry 270°	1.6%	−0.3%
	20×20	Total	0.9%	−0.7%
		Gantry 0°	2.8%	−0.7%
		Gantry 90°	2.0%	0.3%
		Gantry 180°	−5.3%	−0.5%
		Gantry 270°	0.9%	−1.5%
IMRT	10×10	Total	2.0%	0.5%
	20×20	Total	2.6%	1.6%
VMAT	10×10	Total	0.8%	0.1%
	20×20	Total	0.8%	0.4%

Central axis dose agreement using the cubic target. The impact of angular correction is demonstrated.

**Table 2:** Gamma analysis of phantom measurements.

		γ (2%/2 mm, 80%)		γ (3%/3 mm, 80%)	
Plan	Target	No Φ	With Φ	No Φ	With Φ
FFB	10×10	69.5%	100%	98.8%	100%
	20×20	100%	100%	100%	100%
IMRT	10×10	89%	100%	99.6%	100%
	20×20	70%	73%	51%	99.6%
VMAT	10×10	83%	100%	100%	100%
	20×20	61.2%	83.9%	91.3%	99.6%

Gamma analysis for the cubic target plans with focus on the high dose (80% threshold) region. The impact of angular correction is demonstrated.

**Figure 6:**
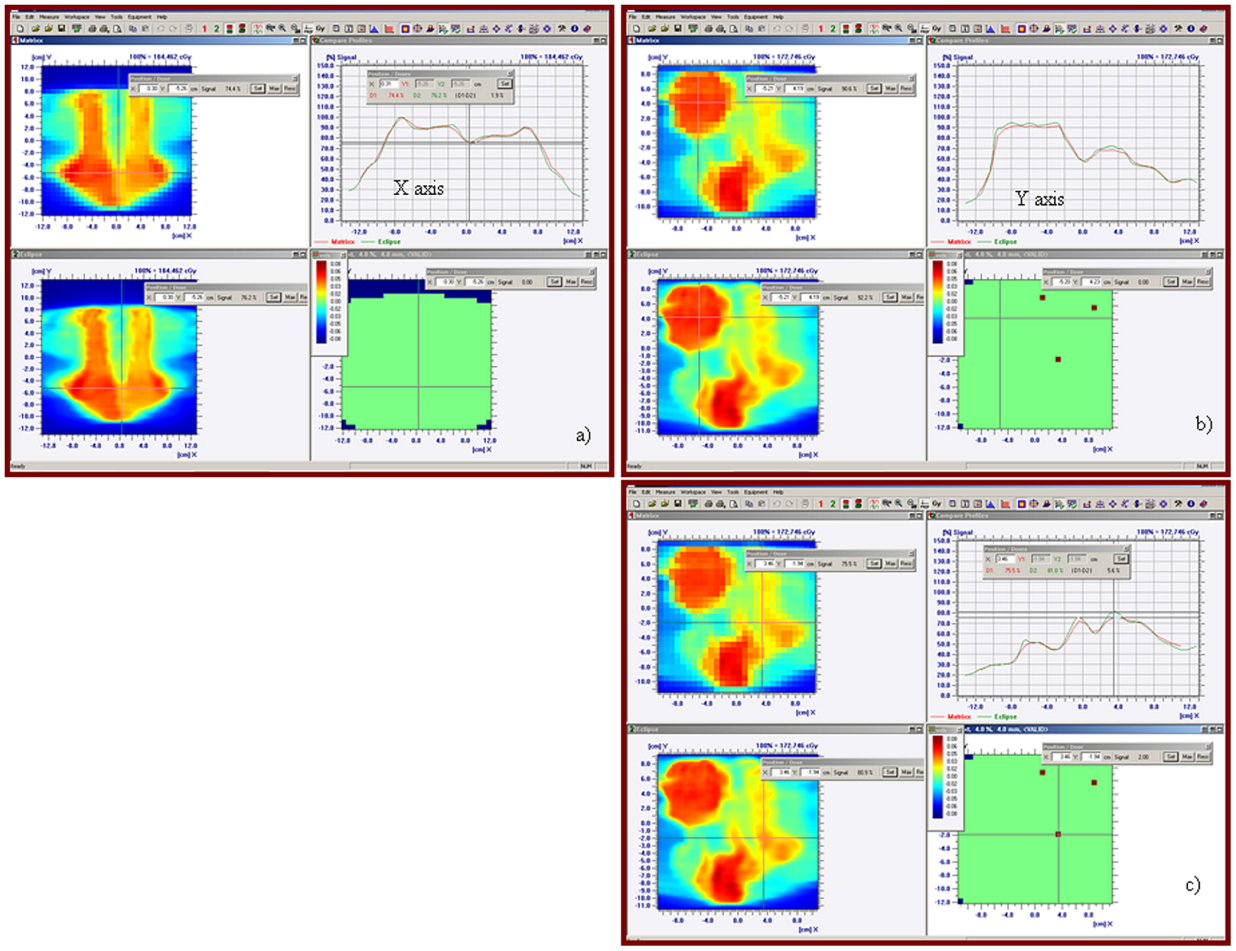
Patient plans are routinely evaluated in the a) coronal and b) sagittal planes, examining dose profiles through areas corresponding to target and critical normal structures. Evaluation of γ(4 mm/4%,10%) >1 is used to highlight pixels requiring further attention. A failing pixel due to an out of plane dose gradient that is large compared to the chamber dimension is illustrated in c.

Coronal and sagittal plane measurements for a head and neck patient plan are illustrated in figure 6. Unlike the phantom plans, patient plans may have substantial dose gradients perpendicular and parallel to the detector array plane. These gradients decrease the accuracy possible with the measurement system dependent upon the characteristics (e.g. size) of the detectors. An example of this is illustrated in figure 6c. Results of gamma analysis for simple (table 3) and complex (table 4) patient plans demonstrate average pass (γ≤1) rates of 99% vs 98.8% and 98.9% vs 98.7% for coronal vs saggital planes in simple and complex plans respectively for γ(3%/3 mm, 10%) with use of angular correction (Φ). Average difference in pass rate was less than 0.8% and 3.2% for simple and complex plans, respectively. However differences as large as 18% were noted for some plans. Minimum pass rate was ≥95.9% vs. 81.6% for angular vs. no angular correction. In practice γ(4%/4 mm, 10%) is used only to highlight areas needing direct examination; it is worth noting that differences in the mean passing rates for the two conditions were within 1.1% and 0.3% for complex and simple plans, respectively.

**Table 3:** Gamma analysis on patient plans of simple targets for full and partial (*) arc plans.

Site	Arcs	Start/Stop	Plane	γ(3%/3 mm, 10%)			γ(4%/4 mm, 10%)		
				With Φ	No Φ	Diff	With Φ	No Φ	Diff
Prostate	2	181–179	Coronal	99.9%	99.5%	0.4%	100.0%	99.9%	0.1%
		179–181	Sagittal	99.9%	100.0%	−0.1%	99.9%	100.0%	−0.1%
Prostate*	1	150–210	Coronal	99.6%	97.9%	1.8%	100.0%	99.7%	0.3%
		150–210	Sagittal	97.9%	96.9%	1.0%	99.7%	99.7%	0.0%
Prostate	2	181–179	Coronal	100.0%	99.9%	0.1%	100.0%	100.0%	0.0%
		179–181	Sagittal	100.0%	99.6%	0.4%	100.0%	100.0%	0.0%
Prostate	2	181–179	Coronal	99.1%	98.7%	0.4%	99.9%	99.9%	0.0%
		179–181	Sagittal	100.0%	99.9%	0.1%	100.0%	100.0%	0.0%
Prostate	2	181–179	Coronal	99.8%	98.2%	1.6%	100.0%	99.8%	0.2%
		179–181	Sagittal	100.0%	99.8%	0.2%	100.0%	99.9%	0.1%
		Min	Coronal	99.1%	97.9%	0.1%	99.9%	99.7%	0.0%
		Max		100.0%	99.9%	1.8%	100.0%	100.0%	0.3%
		Mean		99.7%	98.8%	0.8%	100.0%	99.9%	0.1%
		Stdev		0.3%	0.9%	0.8%	0.0%	0.1%	0.1%
		Min	Sagittal	97.9%	96.9%	−0.1%	99.7%	99.7%	−0.1%
		Max		100.0%	100.0%	1.0%	100.0%	100.0%	0.1%
		Mean		99.6%	99.2%	0.3%	99.9%	99.9%	0.0%
		Stdev		1.0%	1.3%	0.4%	0.1%	0.1%	0.1%

**Table 4:** Gamma analysis on patient plans of complex targets for full and partial (*) arc plans.

Site	Arcs	Arc Start/Stop	Plane	γ(3%/3 mm, 10%)			γ(4%/4 mm, 10%)		
				With Φ	No Φ	Diff	With Φ	No Φ	Diff
RT Neck*	4	270–90/90–270	Coronal	96.4%	83.2%	13.2%	99.8%	87.3%	12.5%
		90–270/270–90	Sagittal	94.7%	93.0%	1.8%	98.9%	96.9%	2.1%
Pancreas	2	179–181	Coronal	99.7%	99.8%	−0.1%	100.0%	99.8%	0.2%
		181–179	Sagittal	98.6%	99.1%	−0.5%	100.0%	100.0%	0.0%
RT Lung	2	181–179	Coronal	99.1%	96.9%	2.2%	100.0%	99.6%	0.4%
		179–181	Sagittal	99.3%	99.0%	0.3%	99.8%	99.9%	−0.1%
Lung*	2	330–179	Coronal	95.9%	95.8%	0.1%	99.3%	99.0%	0.3%
		179–330	Sagittal	99.0%	96.7%	2.3%	100.0%	99.0%	1.0%
Para Aortics	2	179–181	Coronal	97.5%	96.7%	0.8%	99.8%	99.6%	0.2%
		181–179	Sagittal	98.5%	99.5%	−1.0%	99.8%	99.8%	0.0%
Pelvis	3	181–179	Coronal	99.1%	97.3%	1.9%	100.0%	99.8%	0.2%
		179–181/181–179	Sagittal	99.6%	99.1%	0.5%	100.0%	99.9%	0.1%
Pelvis	3	181–179	Coronal	98.9%	94.9%	4.0%	100.0%	99.6%	0.4%
		179–181/181–179	Sagittal	99.9%	99.9%	0.0%	100.0%	100.0%	0.0%
Pelvis	3	181–179	Coronal	98.6%	97.6%	1.1%	100.0%	100.0%	0.0%
		179–181/181–179	Sagittal	98.5%	98.7%	−0.2%	99.9%	99.9%	0.0%
Pelvis	3	181–179	Coronal	99.5%	98.0%	1.6%	99.8%	99.5%	0.3%
		179–181/181–179	Sagittal	99.4%	99.4%	0.0%	99.7%	100.0%	−0.3%
LT Pelvis	2	181–179	Coronal	98.8%	98.4%	0.4%	99.6%	99.5%	0.1%
		179–181	Sagittal	99.3%	99.4%	−0.1%	99.9%	99.9%	0.0%
RT Lung*	2	179–350	Coronal	99.0%	99.4%	−0.4%	100.0%	100.0%	0.0%
		350–179	Sagittal	99.7%	99.3%	0.4%	99.9%	99.9%	0.0%
Pelvis	3	179–181	Coronal	99.5%	97.2%	2.3%	99.6%	99.6%	0.0%
		181–179/179–181	Sagittal	99.7%	99.2%	0.5%	99.9%	99.8%	0.1%
RT Lung-SBIMRT*	2	181–40	Coronal	100.0%	99.5%	0.5%	100.0%	100.0%	0.0%
		40–181	Sagittal	98.4%	100.0%	−1.6%	100.0%	100.0%	0.0%
RT Lung-SBIMRT*	3	181–50	Coronal	100.0%	100.0%	0.0%	100.0%	100.0%	0.0%
		50–181	Sagittal	98.7%	99.7%	−1.0%	99.9%	100.0%	−0.1%
RT Axilla*	2	181–30	Coronal	99.3%	99.1%	0.2%	100.0%	99.8%	0.2%
		30–181	Sagittal	92.4%	86.2%	6.2%	98.1%	93.9%	4.2%
RT Head & Neck*	3	190–170/170–190	Coronal	99.5%	88.4%	11.1%	100.0%	98.6%	1.4%
		230–50	Sagittal	99.2%	97.3%	2.0%	100.0%	99.6%	0.4%
Mediastinum*	3	280–80/80–280	Coronal	99.7%	81.6%	18.1%	100.0%	91.5%	8.5%
		280–80	Sagittal	99.5%	97.7%	1.9%	99.9%	99.9%	0.0%
Brain Boost*	4	181–300/300–181	Coronal	98.9%	95.4%	3.5%	100.0%	99.5%	0.5%
		60–179/179–60	Sagittal	100.0%	100.0%	0.0%	100.0%	100.0%	0.0%
Brain Boost*	4	181–300/300–181	Coronal	100.0%	99.8%	0.2%	100.0%	100.0%	0.0%
		60–179/179–60	Sagittal	100.0%	99.9%	0.1%	100.0%	100.0%	0.0%
									
		Min	Coronal	95.9%	81.6%	−0.4%	99.3%	87.3%	0.0%
		Max		100.0%	100.0%	18.1%	100.0%	100.0%	12.5%
		Mean		98.9%	95.7%	3.2%	99.9%	98.6%	1.3%
		Stdev		1.1%	5.4%	5.2%	0.2%	3.3%	3.3%
									
		Min	Sagittal	92.4%	86.2%	−1.6%	98.1%	93.9%	−0.3%
		Max		100.0%	100.0%	6.2%	100.0%	100.0%	4.2%
		Mean		98.7%	98.1%	0.6%	99.8%	99.4%	0.4%
		Stdev		1.9%	3.3%	1.7%	0.5%	1.5%	1.1%

**Figure 7:**
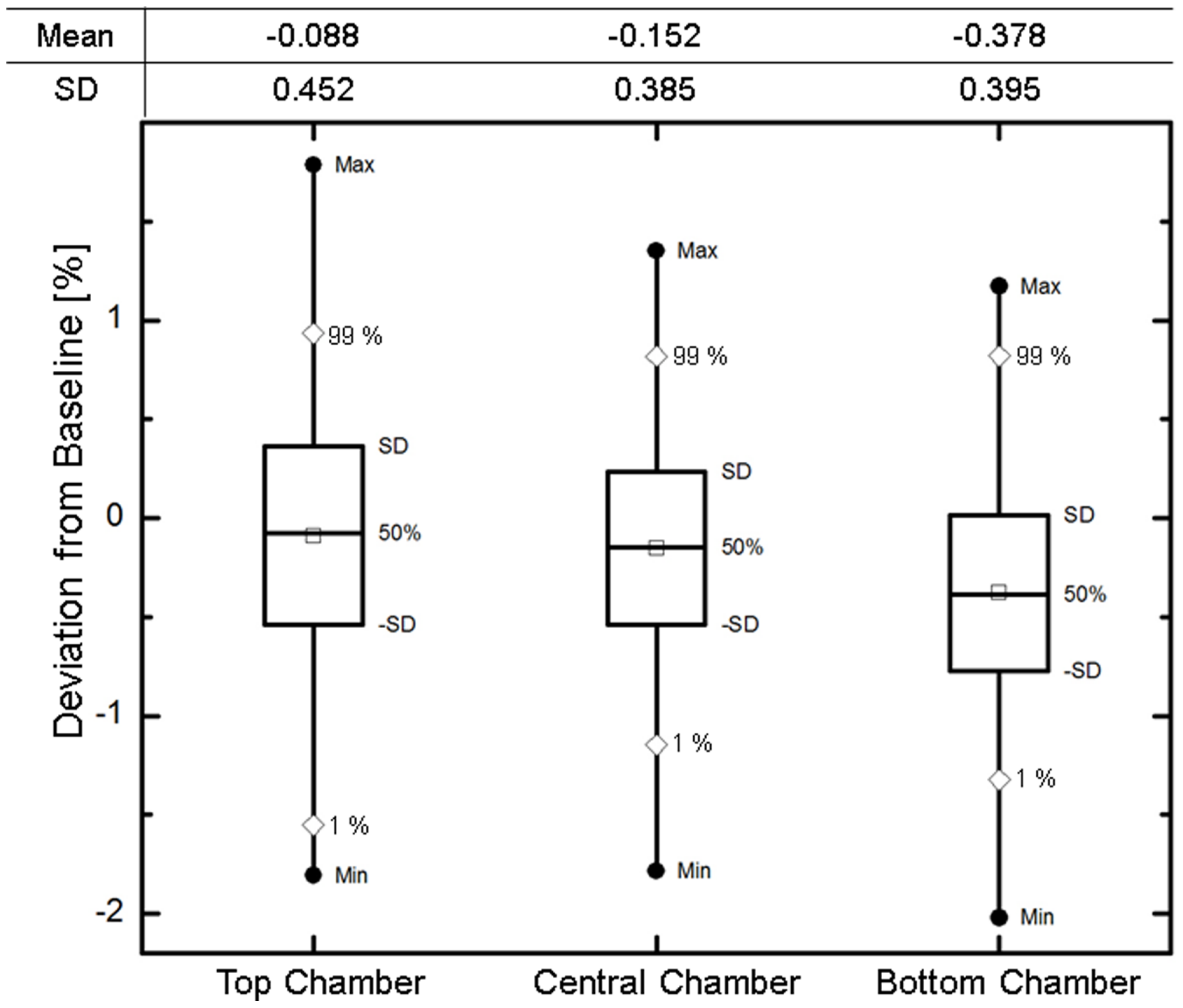
Summary of the daily QA measurement statistics from April 2010 to September 2011 for each of the three chambers utilized in the daily QA device – TRACKER (model 90100). Mean difference from baseline was −0.1%±0.5%, −0.2%±0.4%, −0.4%±0.4% for the top, center and bottom chambers, respectively.

Statistics on daily QA measurements are illustrated in figure 7. The measurements were collected over a time period of nine months and confirm the stability of the system. The original tolerance, based on experience with fixed-gantry DMLC daily QA test, was set to 5% for failure. Based on this data, tolerance can be brought to 3% for failure and a 1.5% for warning while still avoiding any false negatives for VMAT daily QA with a 99% confidence. Special attention was taken during implementation of this test in order to make it part of the morning QA workflow for the therapists. The therapist group confirmed that it was neither disruptive nor time consuming.

## Discussion

Control points tests form an important basis for VMAT commissioning, providing a valuable initial benchmark on mechanical function that may be used as a basis for routine QA testing. Recent studies questioning the value of single field IMRT dosimetry, as well as the advent of VMAT delivery has provided motivation to place the detector array in a volumetric phantom, not coupled to the rotation of the gantry [13]–[15]. The discussed approach enables end-to-end testing with results directly analogous to patient dose measurements. Dose-based tests provide the most direct demonstration of function and accuracy as it pertains to patient plans. In combination with the more common approach of averaging γ results for a sampling of patient plans, the use of a simple cubic target, i.e. FFB, within the phantom used for per-patient measurements provides for simple and reliable benchmarking of the accuracy of the measurement system to quantify differences due to use of VMAT or IMRT. In contrast to other methodologies [9]–[12] the present approach allows for plans with both full and partial arcs, utilizing the full angular range including the couch. Furthermore, it has been demonstrated that target dose-based techniques can easily be extended to daily QA measurements for VMAT.

Angular correction, mean pass rates of 99.7%±0.7% for simple plans and 98.8%±1.5% for the complex plans sampled, compared favorably with other reported results. In practice, we do not use γ as a sufficient statistic for defining acceptability of a patient’s VMAT plan owing to vagaries in definition of an absolute threshold that may be meaningfully and universally connected to specific details about the dose distribution^(13,14)^. Task Group 119 finds from their results that γ <1 with 3%/3 mm should be >88% for 95% of the plans examined but does not indicate absolute minimum level of acceptability. Also, authors differ in use and reporting of requirement of a minimum threshold dose for pixels included in the γ calculation. Calculation of central axis dose accuracy for a simple, standardized target volume adds a useful benchmark.
